# The Role of Anxiety and Prosocial Behaviors on Adherence Behaviors to Prevent COVID-19 in University Students in the United States: Cross-Sectional Study

**DOI:** 10.2196/52970

**Published:** 2024-05-27

**Authors:** Silvia Corbera, Amanda M Marín-Chollom

**Affiliations:** 1 Department of Psychological Science, Central Connecticut State University, New Britain, CT, United States

**Keywords:** prosocial behavior, COVID-19, anxiety, COVID-19 prevention, preventive health behavior, adherence to prevention

## Abstract

**Background:**

In situations of acute stress, individuals may engage in prosocial behaviors or risk-taking self-oriented behaviors. The COVID-19 pandemic created large stress-promoting conditions that impacted individuals’ decisions to adhere to COVID-19 preventative behaviors.

**Objectives:**

The study aimed to examine the relationship between anxiety during the pandemic and adherence behaviors to prevent the spread of COVID-19, and the moderating influence of prosocial behaviors. We hypothesized that individuals with high anxiety during COVID-19 would adhere more to preventive COVID-19 behaviors than ones with low anxiety and that this relationship would be stronger in those individuals with higher prosocial behaviors.

**Methods:**

A web-based survey was administered through the SONA web-based participant tool of the psychology department of a university in the Northeastern United States. A final sample of 54 undergraduate students completed web-based questionnaires during the second wave of the COVID-19 pandemic, from January to May 2021, which included demographic measures and surveys on prosocial behaviors, anxiety, and COVID-19 preventive behaviors. Moderation analyses were conducted using PROCESS in SPSS.

**Results:**

Participants reported high levels of trait and state anxiety symptoms, most of them meeting or exceeding the cutoff criteria to be clinically meaningful (state anxiety: 47/54, 87%; trait anxiety: 38/44, 86%), and over 50% highly adhered to the COVID-19 preventive behaviors of wearing a face mask, using hand sanitizer, handwashing, coughing/sneezing into their elbow or a tissue, self-quarantining, maintaining social distance, avoiding social gatherings, and avoiding nonessential travel. No significant associations were observed between prosocial behavior, anxiety types, and adherence to COVID-19 preventive behaviors. However, when moderation analyses were conducted between anxiety types and adherence to COVID-19 preventive behaviors, results demonstrated a statistically significant interaction of public prosocial behavior with state anxiety (β=−.17, *t*_53_=−2.60; *P*=.01), predicting engagement in COVID-19 preventative behaviors. At high levels of anxiety, low levels of prosocial public behaviors were associated with higher engagement in COVID-19 preventative behaviors. In contrast, high levels of public prosocial behavior were associated with low engagement in COVID-19 preventative behaviors at higher levels of anxiety.

**Conclusions:**

These results provide information that can aid in the creation of interventions that could increase adherence to COVID-19 preventative behaviors (Reviewed by the Plan P #PeerRef Community).

## Introduction

The understanding of human behavior under stress and in situations of anxiety has been an extensive topic of research [1,2]. Ample evidence suggests that acute stress affects general cognition, executive functioning, working memory [3-5], social-emotional information processing, and social and prosocial behavior [6-8]. Stress also affects decision-making skills [1,2,9-11]; however, this relationship depends on several factors, such as the type of decision-making situation [9], time pressure [11], and the individual characteristics of the participants, such as gender [2,12].

Evidence has also shown that social behavior and social decision-making can be influenced by stress; however, studies are conflicted in this area. Some studies have shown that in situations of acute stress, individuals might engage in more prosocial and empathetic behaviors such as prosocial trustworthiness and sharing [4,7,13,14]; however, on the other hand, other studies suggest that in situations of stress, individuals might engage in unfavorable and risk-taking self-oriented behaviors and distrust [15,16]. Most of the research on the effects of stress in decision-making has been conducted in laboratory studies using stress-inducing paradigms, in which the stress induced was either physical (eg, cold pressure test in which participants immerse their hand in a bucket of cold water) or psychosocial (eg, Trier Social Stress Test in which participants perform a mock job interview). However, to our knowledge, just one study has investigated decision-making in the context of the COVID-19 pandemic as the stressor [17]. Romero-Rivas and Rodriguez-Cuadrado [17] examined whether the psychological impact generated by the COVID-19 pandemic influenced decision-making processes by presenting participants with four decision-making tasks (the dictator game, framing problems, utilitarian/deontological moral dilemmas, and altruistic/egoistic moral dilemmas). This study showed that higher levels of psychological impact were related to safer responses in framing problems and more deontological/altruistic responses to moral dilemmas [17], suggesting that the psychological impact of COVID-19 affected decision-making processes by participants showing safer and more altruistic responses using laboratory decision-making tasks. Given the influence of stress in the decision-making process [1,2], it is imperative to understand this relationship further, especially during the COVID-19 pandemic as it can aid in understanding the factors that influence compliance and adherence to behaviors to prevent the spread of COVID-19 and its variants.

The worldwide COVID-19 pandemic placed humanity in a devastating global health challenge that required the population to adapt to a rapidly changing situation. Gruber and colleagues [18] conceptualized the COVID-19 pandemic as a multidimensional complex stressor that affected both the individual and family, and multiple societal layers, with toxic social stressors such as social isolation and financial loss [18]. Stress research has shown that situations that are uncontrollable and uncertain, and have a social evaluative threat elicit high stress responses [19]. The COVID-19 pandemic created large stress-promoting conditions, such as uncertainty, life threat, loss, and long exposure to anxiety-inducing information [18], generating extensive detrimental psychological and mental health consequences [18,20]. A recent meta-analysis by Luo and colleagues [20] reported a prevalence of anxiety of 33% and depression of 28% of the population during the COVID-19 pandemic, and a study by Ebrahimi and colleagues [21] reported that the prevalence of clinical anxiety increased 2-3 times during the COVID-19 pandemic in comparison to estimations of similar samples before the pandemic. In addition, the COVID-19 pandemic has increased the prevalence of anxiety among undergraduate college students [22,23], especially among the lower socioeconomic groups [22].

To prevent the spread of COVID-19, public health measures were introduced that involved engagement in new behaviors, and the population was expected to adhere to these preventive behaviors (eg, limiting social distance and using masks). Decisions made under different levels of risk and ambiguity can be affected differently by stress [9,24], and during the stress-promoting conditions inherent in the COVID-19 pandemic, the population was confronted with making high-risk decisions about their behaviors, especially decisions related to preventing the spread of COVID-19.

A few studies have examined the predictors of adherence to preventive behaviors to spread COVID-19 [21,25-27]. Recent research by Pollak and colleagues [25] showed that predictors for nonadherence were high levels of current distress and risk factors such as male sex, not having children, attention-deficit/hyperactivity disorder symptoms, smoking, and high levels of risk-taking behavior. Similarly, Ebrahimi and colleagues [21] reported that female sex, older age, worry about significant others, mandatory adherence, and altruistic attitude were associated with higher adherence, and current employment was associated with lower adherence. Besides, altruistic attitudes, empathy, fairness, and gratitude have also been associated with higher levels of adherence to COVID-19 preventative behaviors [21,26,27].

To our knowledge, no studies have examined the relationship between anxiety and adherence to behaviors to prevent the spread of COVID-19 with the moderating role of prosocial tendencies. Therefore, this study aims to investigate the relationship between anxiety during the COVID-19 pandemic and adherence behaviors to prevent the spread of COVID-19, and the moderating influence of prosocial tendencies on this association.

It is hypothesized that individuals with high anxiety during the COVID-19 pandemic are more likely to adhere to the behaviors to prevent the spread of COVID-19 than individuals with low anxiety, and this relationship will be stronger for individuals with high prosocial behavior tendencies than those with low prosocial behavior tendencies.

## Methods

### Participants

Participants were undergraduate students recruited at a university in the Northeastern United States through the psychology department’s web-based subject pool that is managed by SONA. Students from different majors taking psychology courses were eligible to sign up to participate in this study and received credit in their courses for participation. Data was collected on the web via Select Survey in the Spring 2021 semester during the COVID-19 pandemic from January 2021 to May 2021. In addition to the measures described below, basic demographics of age, race/ethnicity, class standing, gender, and employment status were also collected.

### Ethical Considerations

This study was approved by Central Connecticut State University’s Institutional Review Board (IRB#20089), and web-based consent was obtained from all participants. Participants were presented with a web-based informed consent, and they acknowledged it by pressing a button to continue the study and before any data collection. Participants were informed that their information would be kept confidential, and their data would be anonymous. Participants were informed that their data would be part of scientific publications. They were granted 1 SONA credit for 15-30 minutes of their time to complete the questionnaire.

### Measures

#### Prosocial Behaviors

The 23-item Prosocial Tendencies Measure [28,29] was developed to be used with college-age students and young adults, and was used to measure six types of prosocial behaviors: compliant (2 items), dire (3 items), altruistic (5 items), public (4 items), emotional (4 items), and anonymous (5 items). Compliant is when prosocial behaviors are done because others asked for help (eg, “I never hesitate to help others when they ask for it”). Dire is when prosocial behaviors are done to help others in emergencies or crises (eg, “I tend to help people who hurt themselves badly”). Altruistic is helping others and expecting little to nothing in return (eg, “I often help even if I don’t think I will get anything out of helping”). Public is when prosocial behaviors are completed because others are watching with the motivation to receive external approval (eg, “Helping others when I am in the spotlight is when I work best”). Emotional is when individuals help others because they are in a highly emotional state (eg, “It is most fulfilling to me when I can comfort someone who is very distressed”). Anonymous is when prosocial social behaviors are enacted intentionally without other people’s knowledge (eg, “I tend to help needy others most when they do not know who helped them”). Items were answered on a 5-point Likert Scale from 1 (does not describe me at all) to 5 (describes me greatly). Six total sum subscales were calculated and used in the analysis. Higher scores indicate greater prosocial behavior for each subscale. This scale has shown acceptable to good reliability (public subscale: Cronbach α=0.80; anonymous subscale: Cronbach α=0.88; dire subscale: Cronbach α=0.54; emotional subscale: Cronbach α=0.77; compliant subscale: Cronbach α=0.87; altruism subscale: Cronbach α=0.62) and high test-retest reliability with 2-week reliability correlations (coefficients of public: 0.61; anonymous: 0.75; dire: 0.72; emotional: 0.80; compliant: 0.73; altruism: 0.60; all *P*<.001) [28].

#### Anxiety

The State-Trait Anxiety Inventory was used to measure both trait and state anxiety [30,31]. The 40-item inventory uses 20 items for trait anxiety (eg, “I am a steady person”) and 20 for state anxiety (eg, “I feel at ease”). Of the 40 items, 19 are reverse scored. All items in this study were rated on a 4-point Likert scale from not at all to very much so. Two total sum subscales were calculated, and higher scores indicate greater anxiety for both subscales. Internal consistency coefficients range from 0.86 to 0.95 and test-retest from 0.65 to 0.75 for 2 months [30].

#### COVID-19 Preventative Behaviors

The COVID-19 International Survey from the PhenX Toolkit [32,33] was used to specifically collect data on what preventative COVID-19 behaviors participants were engaging in. For this study, we selected 23 items within the survey specific to what frequency individuals were engaging in COVID-19 preventative behaviors (eg, hand washing, mask wearing, physical distancing, avoiding social gatherings, self-quarantining after travel, or self-quarantining if infected or likely infected). These items are answered on a 4-point Likert scale from never to most of the time, with the additional option of “don’t know/I prefer not to answer/Not applicable.” One total sum score was calculated, and higher scores indicated higher engagement in COVID-19 preventative behaviors.

### Analysis

Bivariate correlations between the main variables of interest were first examined. Moderation analyses were then conducted using PROCESS version 3.5 in SPSS Version 29 (IBM Corp). Bootstrapping was set to 5000 and the CI to 95%. Mean-deviated predictor variables (ie, prosocial behaviors and anxiety types) were created and used for all moderation analyses. A total of 12 separate models were tested. One for each type of the six prosocial behaviors, interacting with the two subtypes of anxiety measured that predict engagement in COVID-19–related preventative behaviors. All significant interactions were plotted, using 1 SD below and above the mean for prosocial behaviors, for interpretation. The parameters for using moderation in PROCESS are like any moderation analysis program [34].

## Results

Participants were (n=54) college students (mean age 20.74, SD 5.16, range 18-54 years; 1 student was 54 years of age). Most students’ ages were closer to the mean (25% percentile: n=19; percentile 50%: n=19; percentile 75%: n=21). Students were mostly enrolled full-time at the university (n=43); employed (n=43); and living at home with their parent, relative, or guardian (n=40). Most of the sample self-identified as women (n=43; men: n=9; transgender: n=1) and White (n=34; Hispanic/Latino/a: n=11; Black/African American: n=7; Asian: n=2). See Table 1 for more demographic information.

**Table 1.** Demographic characteristics.

Variable		Participants (N=54), n (%)
**Gender**		
	Women	43 (80)
	Men	9 (17)
	Transgender	1 (2)
**Race/ethnicity**		
	White	34 (63)
	Hispanic/Latino/a	11 (20)
	Black/African American	7 (13)
	Asian	2 (4)
**Enrollment status**		
	Full-time	43 (80)
	Part-time	10 (20)
**First generation college student**		
	Yes	22 (41)
	No	32 (59)
**Marital status**		
	Single	40 (74)
	In a relationship	13 (24)
	Married	1 (2)
**Employed**		
	Yes	43 (80)
	No	10 (20)
**Hours worked per week**		
	<5	4 (9)
	5-10	2 (4)
	10-20	17 (37)
	20-30	17 (37)
	>30	6 (13)
**Housing**		
	On campus (residence hall)	8 (15)
	Off-campus housing (within 5 miles of campus)	6 (11)
	Off-campus housing (farther than 5 miles from campus)	39 (72)
**Living situation**		
	Living alone	5 (9)
	Living with students	5 (9)
	Living with parents/guardians/relatives	40 (74)
	Living with spouse	4 (7)

Participants on average reported high levels of anxiety symptoms on both the trait (mean 51.05, SD 10.37) and state subscales (mean 50.63, SD 11.11). Using the recommended cutoff of 40 to determine clinically meaningful anxiety symptomology, most of the sample either met or exceeded the criteria (state anxiety: 47/54, 87%; trait anxiety: 38/44, 86%). On average, participants highly adhered to COVID-19 preventative actions and behaviors (mean 60.28, SD 13.29). Over 50% of the sample (N=54) engaged in the following behaviors “most of the time”: wearing a face mask (n=49, 91%), using hand sanitizer (n=45, 83%), handwashing with soap and water (n=44, 82%), coughing/sneezing into their elbow (n=44, 82%), self-quarantining if they have or believe they have the virus (n=42, 78%), self-quarantining if they are returning from a trip (n=34, 63%), coughing/sneezing into a tissue and throwing it away and washing hands (n=37, 69%), staying 6 feet apart from other people (n=29, 54%), avoiding large social gatherings (n=29, 54%), and avoiding any nonessential travel (n=29, 54%; Table 2).

**Table 2.** Engagement in COVID-19 preventative behaviors in the past 7 days as reported by participants (N=54).

Action or behavior	Participants responding “most of the time,” n (%)
Wearing a face mask	49 (91)
Use hand sanitizer	45 (83)
Handwashing with soap and water	44 (82)
Coughing/sneezing into your elbow	44 (82)
Self-quarantining if you have or believe you have the virus	42 (78)
Coughing/sneezing into a tissue throwing it away and wash hands	37 (69)
Self-quarantining if you are returning from a trip	34 (63)
Staying 6 feet apart from other people	29 (54)
Avoid large social gatherings	29 (54)
Avoiding any nonessential travel	29 (54)
Avoiding going out to bars/pubs	26 (48)
Avoiding using public transportation	24 (44)
Staying/working at home	21 (39)
Avoiding playdates	16 (30)
Exercise outside alone or with people you live with only	16 (30)
Avoiding going to restaurants	12 (22)
Avoiding taking your children to the park	11 (20)
Avoiding all social gatherings (large and small)	10 (19)
Wearing gloves every time you go out of your home	6 (11)
Avoiding going to the grocery store or pharmacy	6 (11)
Avoiding opening the mail or delivered goods	6 (11)
Avoiding getting take-out food or delivery	5 (9)
Avoiding going for walks	1 (2)

**Figure 1.**
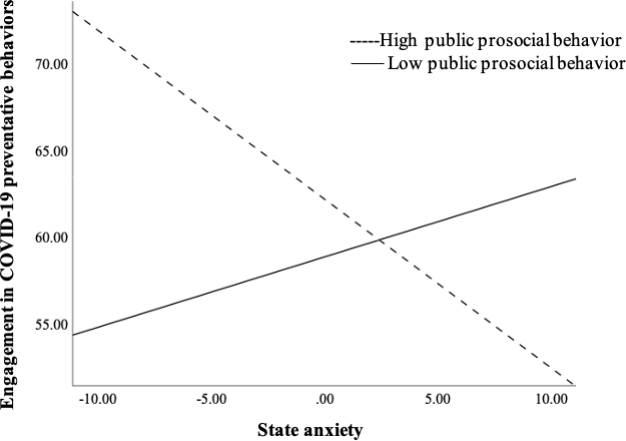
Public prosocial behavior moderating the association between state anxiety and engagement in COVID-19 preventative behaviors.

None of the prosocial behavior types or anxiety types were associated with engagement in COVID-19 preventative behaviors (see Table 3 for descriptive and correlations of study variables). However, in the one model tested with public prosocial behavior, the interaction of public prosocial behavior with state anxiety predicting engagement in COVID-19 preventative behaviors was statistically significant (β=−.17; *P*=.01), which suggested a crossover effect (Figure 1).

**Table 3.** Descriptive statistics and correlations for study variables.

Variable		Participants, n	Mean (SD)		1	2	3	4	5	6	7	8	9
**1. COVID-19 PB** ^**a**^		54	60.28 (13.29)									
*r*			—^b^	0.06	0.01	–0.16	0.04	–0.03	0.16	–0.03	–0.01
	*P* value				—	.66	.93	.26	.78	.85	.26	.87	.97
**2. Public PT** ^**c**^		54	8.26 (3.48)									
*r*			0.06	—	–0.11	–0.78	–0.05	–0.26	0.26	–0.03	0.05
	*P* value				.66	—	.41	<.001	.75	.05	.06	.84	.74
**3. Emotional PT**		54	13.93 (3.33)									
*r*			0.01	–0.11	—	0.09	0.64	0.64	0.49	–0.16	–0.21
	*P* value				.93	.41	—	.53	<.001	<.001	<.001	.31	.12
**4. Altruistic PT**		54	19.83 (4.15)									
*r*			–0.16	–0.78	0.09	—	0.02	0.31	–0.20	–0.06	–0.09
	*P* value				.26	<.001	.53	—	.86	.02	.14	.72	.53
**5. Dire PT**		54	10.35 (2.63)									
*r*			0.04	–0.05	0.64	0.02	—	0.64	0.55	0.03	0.11
	*P* value				.78	.75	<.001	.86	—	<.001	<.001	.84	.43
**6. Compliant PT**		54	7.89 (1.76)									
*r*			–0.03	–0.26	0.64	0.31	0.64	—	0.38	–0.23	–0.20
	*P* value				.85	.05	<.001	.02	<.001	—	.005	.14	.14
**7. Anonymous PT**		54	14.74 (4.34)									
*r*			0.16	0.26	0.49	–0.20	0.55	0.38	—	–0.13	–0.18
	*P* value				.26	.06	<.001	.14	<.001	.005	—	.42	.19
**8. Trait anxiety**		44	51.05 (10.37)									
*r*			–0.03	–0.03	–0.16	–0.06	0.03	–0.23	–0.13	—	0.84
	*P* value				.87	.84	.31	.72	.84	.14	.42	—	<.001
**9. State anxiety**		54	50.63 (11.11)									
	*r*				–0.01	0.05	–0.21	–0.09	0.11	–0.20	–0.18	0.84	—
	*P* value				.97	.74	.12	.53	.43	.14	.19	<.001	—

a PB: preventative behaviors.

b Not applicable.

c PT: prosocial tendency.

At low levels of prosocial public behaviors, engagement in COVID-19 preventative behavior increases as anxiety increases; in contrast, at high levels of prosocial public behaviors, engagement in COVID-19 preventative behavior decreases as anxiety increases. In the other 11 models tested, none of the interaction effects with prosocial behaviors and anxiety-predicting adherence to COVID-19 preventative behavior were statistically significant. (Tables4  5).

**Table 4.** Association between state anxiety and COVID-19 preventative behaviors moderated by six types of prosocial behaviors (N=54).^a^

Model		Estimate (SE)		t test (*df*)	*P* value
**Complaint**		−0.58 (1.12)		−0.52 (53)	.61
	SA^b^	−0.88 (0.75)		−1.18 (53)	.25
	Complaint × SA	0.13 (0.11)		1.19 (53)	.24
**Emotional**		−0.25 (0.60)		−0.42 (53)	.68
	SA	−0.78 (0.55)		−1.42 (53)	.16
	Emotional × SA	0.07 (0.05)		1.49 (53)	.14
**Public**		0.41 (0.51)		0.80 (53)	.43
	SA	−0.33 (0.20)		–1.65 (53)	.10
	Public × SA	0.17 (0.06)		−2.60 (53)	.01
**Altruistic**		−0.60 (0.44)		−1.38 (53)	.17
	SA	−1.76 (0.94)		−1.88 (53)	.07
	Altruistic × SA	−0.10 (0.05)		1.88 (53)	.07
**Dire**		0.31 (0.73)		0.42 (53)	.68
	SA	−0.33 (0.48)		−0.69 (53)	.49
	Dire × SA	0.04 (0.06)		0.71 (53)	.50
**Anonymous**		0.51 (0.44)		1.16 (53)	.25
	SA	−0.16 (0.42)		−0.37 (53)	.71
	Anonymous × SA	−0.02 (0.04)		−0.48 (53)	.63

a Dependent variable: COVID-19 preventative behaviors. All variables were mean deviated.

b SA: state anxiety.

**Table 5.** Association between trait anxiety and COVID-19 preventative behaviors moderated by the six types of prosocial behaviors (n=44).^a^

Model		Estimate (SE)		t test (*df*)	*P* value
**Complaint**		−0.78 (1.24)		−0.63 (43)	.53
	TA^b^	−0.75 (0.86)		−0.88 (43)	.39
	Complaint × TA	0.11 (0.13)		0.83 (43)	.42
**Emotional**		−0.25 (0.69)		−0.37 (43)	.72
	TA	−0.16 (0.66)		−0.24 (43)	.81
	Emotional × TA	0.01 (0.06)		0.18 (43)	.86
**Public**		0.35 (0.63)		0.55 (43)	.58
	TA	−0.27 (0.24)		−1.13 (43)	.26
	Public × TA	−0.14 (0.07)		−1.88 (43)	.07
**Altruistic**		−0.56 (0.53)		−1.06 (43)	.30
	TA	−1.15 (1.11)		–1.04 (43)	.30
	Altruistic × TA	0.06 (0.06)		1.02 (43)	.31
**Dire**		−0.31 (0.87)		−0.36 (43)	.72
	TA	−0.62 (0.78)		−0.79 (43)	.43
	Dire × TA	0.07 (0.08)		0.78 (43)	.44
**Anonymous**		0.33 (0.54)		0.61 (43)	.55
	TA	0.18 (0.49)		0.36 (43)	.72
	Anonymous × TA	−0.02 (0.04)		−0.44 (43)	.66

a Dependent variable: COVID-19 preventative behaviors. All variables were mean deviated, except the dependent variable.

b TA: trait anxiety.

## Discussion

### Principal Findings

This study aimed to examine the association between state and trait anxiety during the COVID-19 pandemic and the adherence behaviors to prevent the spread of COVID-19 (eg, handwashing and mask wearing), and to investigate the moderating role of prosocial behaviors on this association. Results revealed a statistically significant interaction of public prosocial behaviors with state anxiety predicting adherence to COVID-19 preventative behaviors. This interaction showed that only at low levels of prosocial behaviors (ie, lower self-oriented tendencies with less approval-seeking tendencies), as anxiety increased, engagement in COVID-19 preventative behavior increased, and in contrast, at high levels of prosocial public behaviors (ie, higher self-oriented tendencies with more approval-seeking tendencies), as anxiety increased, adherence to COVID-19 preventative behavior decreased. More specifically, our results revealed that there were no associations between state and trait anxiety and any of the prosocial behaviors (emotional, altruism, dire, anonymous, public, and compliant), and only public prosocial tendencies showed a significant interaction with state anxiety, predicting engagement in COVID-19 preventative behaviors, and suggesting a crossover effect.

### Comparison With Prior Work

Based on previous literature on decision-making under stress-promoting conditions [4,7,13,14], we hypothesized that participants with high state and trait anxiety would be more likely to adhere to the behaviors to prevent the spread of COVID-19 than those with low state and trait anxiety, and that this relationship would be stronger for individuals with high prosocial behavior tendencies than those with low prosocial tendencies. Because of the exceptional situation created by the COVID-19 pandemic, we did not specifically predict that any type of prosocial behavior would show a stronger relationship than another.

Concerning the lack of association between state and trait anxiety and any of the prosocial behaviors, these results were not surprising, as we expected that differential levels of prosocial tendencies would relate differently with anxiety when predicting adherence behaviors and therefore that they would operate as a moderator. On the other hand, the interaction that partially supported our hypothesis was with public prosocial behaviors, which moderated the relationship between state anxiety and adherence behaviors to prevent COVID-19. This moderation effect demonstrated a crossover effect. More specifically, at low levels of prosocial public behaviors (ie, lower self-oriented tendencies with less approval-seeking tendencies), as anxiety increases, engagement in COVID-19 preventative behavior increases; in contrast, at high levels of prosocial public behaviors (ie, higher self-oriented tendencies with more approval-seeking tendencies), as anxiety increases, engagement in COVID-19 preventative behavior decreases. As described in the validation study of the Prosocial Tendencies Measure of Carlo and Randall [28], the public prosocial scale was significantly negatively correlated with the altruism, anonymous, and the compliant prosocial subscales. Additionally, public prosocial behaviors were shown to be inversely associated with sympathy, ascription of responsibility, perspective taking, and internalized prosocial moral reasoning, and on the other hand, positively associated with approval-oriented prosocial moral reasoning and hedonism [28]. Therefore, individuals endorsing higher public prosocial tendencies would show more approval-seeking behaviors and be more oriented toward their own needs (as opposed to other people’s needs).

Even though we did not find a direct relationship between altruistic, prosocial, or compliant prosocial behaviors, which are the types that capture other-oriented, sympathetic, and morally oriented behaviors, and anxiety and adherence to COVID-19 preventive behaviors, the results revealed that individuals with low levels of public and other approval-seeking behaviors would be more likely to engage in COVID-19 preventive behaviors in situations with high anxiety, which partially supports our hypothesis. The lack of effects with trait anxiety could be due to the smaller sample size of participants answering the trait anxiety scale (n=44) compared to the larger size (N=54) that completed the state anxiety scale, or state anxiety may be more relevant to the COVID-19 pandemic context as it was situational. Even though both types of anxiety were high in this sample (87% of the sample in state anxiety and 86% of the sample in trait anxiety were above the cutoff of 40), state anxiety may have a bigger influence on prosocial and adherence behaviors within this context.

In addition, given the inverse relationship between public and altruistic prosocial behaviors [28], these results suggest that individuals low on public prosocial behaviors would show more other-oriented behaviors. On the other hand, our study did not find any differential relationship between individuals with altruistic prosocial behaviors and state anxiety and adherence behaviors, and therefore, more research is needed in this area. Our results are in partial agreement with the study of Romero-Rivas and Rodriguez-Cuadrado [17], which found that high states of psychological impact from the COVID-19 pandemic were related to individuals being more risk averse and more altruistic in decision-making. Our results showed higher state anxiety was related to more engagement in adherence behaviors to prevent COVID-19 in only the individuals with lower public prosocial behaviors (more other-oriented and less approval-seeking behaviors). Therefore, we propose that higher anxiety relates to prosocial decision-making differently depending on the individual characteristics, in which the individuals with more other-oriented and less approval-seeking prosocial tendencies with higher state anxiety would be more likely to adhere to COVID-19 preventive behaviors. On the other hand, individuals with higher levels of self-oriented or approval-seeking behaviors (public prosocial) show less adherence to COVID-19 preventive behaviors with an increase in state anxiety. As the group with high public prosocial tendencies has been reported to be motivated by self-oriented and approval-seeking tendencies [28], we speculate that situations with high state anxiety would prevent these individuals from engaging in behaviors that would necessitate other-oriented prosocial behaviors and, therefore, would prevent them from engaging in prosocial decision-making and other-oriented behaviors.

### Strengths and Future Directions

We suggest that more studies are needed to explore the role of each type of prosocial behavior tendency in addition to a global measure of prosocial tendencies in the engagement of adherence behaviors to prevent COVID-19. Additionally, other variables that may have influenced the results such as vaccine hesitancy, perceived personal risk and disease vulnerability, and trust in science may be potential variables to study in future research, especially regarding the factors that may impact the adherence to preventive behaviors in young adults [35,36]. Our results further the current knowledge on the factors that predict the engagement to adherence behaviors to prevent COVID-19 and provide insights on the cultivation and creation of interventions to promote other-oriented versus self-oriented motivations and tendencies, and in the creation of anxiety-reducing interventions that could increase adherence to COVID-19 preventative behaviors.

### Limitations

Our study has several limitations. First, the sample size was small, with 54 participants. However, the performed power analysis, with a power (β) set at 0.8, the Cohen *f* at 0.15 for medium effects, and significance level at .05 (SPSS v29), indicated that the sample needed was 55. Given that the study was conducted during the second wave of the COVID-19 pandemic, we obtained these participants at a critical time point, and we did not recruit additional participants to increase the study sample as the world and life circumstances changed substantially after spring 2021. Second, the sampling method used was convenience sampling with an undergraduate college population, which could have led to the collection of data from participants who were more motivated and self-selected. Additionally, this convenience sampling method led to the recruitment of a homogeneous sample comprised of undergraduate students. This may have impacted the generalizability of the results, and we raise caution when extrapolating results across age groups, educational backgrounds, and cultural contexts. Third, given the cross-sectional design of the study and use of web-based self-reports, we cannot assert causal relationships between the variables of the study, which limits the interpretation of the data. Fourth, because of the small sample size and the cross-sectional nature of the study, these results on their own cannot make any definitive public health policy recommendations but can inform other studies on the role of prosocial tendencies in the relationship between stress and adherence to preventive behaviors. Additionally, the study was conducted on the web, and therefore, it was not possible to control for attention and effort in the participants’ responses. Finally, the measures were self-report questionnaires that are prone to biases such as social desirability.

### Conclusion

To sum up, our results revealed that anxiety was differently associated with prosocial decision-making processes. At low levels of prosocial public behaviors, as state anxiety increased, the engagement in adherence to behaviors to prevent the spread of COVID-19 also increased. On the other hand, at high levels of prosocial public behaviors, as state anxiety increased, the engagement in adherence to behaviors to prevent the spread of COVID-19 decreased. These results provide valuable information that can aid in the creation of interventions to promote other-oriented motivations and in the reduction of anxiety that could increase adherence to COVID-19 preventative behaviors.

## References

[1] Starcke K, Brand M (2012). Decision making under stress: a selective review. Neurosci Biobehav Rev.

[2] Nowacki J, Heekeren HR, Deuter CE (2019). Decision making in response to physiological and combined physiological and psychosocial stress. Behav Neurosci.

[3] Shields GS, Sazma MA, Yonelinas AP (2016). The effects of acute stress on core executive functions: a meta-analysis and comparison with cortisol. Neurosci Biobehav Rev.

[4] Wolf OT, Schulte JM, Drimalla H, Hamacher-Dang TC, Knoch D, Dziobek I (2015). Enhanced emotional empathy after psychosocial stress in young healthy men. Stress.

[5] Wingenfeld K, Duesenberg M, Fleischer J (2018). Psychosocial stress differentially affects emotional empathy in women with borderline personality disorder and healthy controls. Acta Psychiatr Scand.

[6] Frisch JU, Häusser JA, Mojzisch A (2015). The Trier Social Stress Test as a paradigm to study how people respond to threat in social interactions. Front Psychol.

[7] von Dawans B, Strojny J, Domes G (2021). The effects of acute stress and stress hormones on social cognition and behavior: current state of research and future directions. Neurosci Biobehav Rev.

[8] Gonzalez-Liencres C, Breidenstein A, Wolf OT, Brüne M (2016). Sex-dependent effects of stress on brain correlates to empathy for pain. Int J Psychophysiol.

[9] Starcke K, Brand M (2016). Effects of stress on decisions under uncertainty: a meta-analysis. Psychol Bull.

[10] de Visser L, van der Knaap LJ, van de Loo AJAE, van der Weerd CMM, Ohl F, van den Bos R (2010). Trait anxiety affects decision-making differently in healthy men and women: towards gender-specific endophenotypes of anxiety. Neuropsychologia.

[11] Soshi T, Nagamine M, Fukuda E, Takeuchi A (2019). Pre-specified anxiety predicts future decision-making performances under different temporally constrained conditions. Front Psychol.

[12] van den Bos R, Harteveld M, Stoop H (2009). Stress and decision-making in humans: performance is related to cortisol reactivity, albeit differently in men and women. Psychoneuroendocrinology.

[13] von Dawans B, Ditzen B, Trueg A, Fischbacher U, Heinrichs M (2019). Effects of acute stress on social behavior in women. Psychoneuroendocrinology.

[14] von Dawans B, Fischbacher U, Kirschbaum C, Fehr E, Heinrichs M (2012). The social dimension of stress reactivity: acute stress increases prosocial behavior in humans. Psychol Sci.

[15] Bendahan S, Goette L, Thoresen J, Loued-Khenissi L, Hollis F, Sandi C (2017). Acute stress alters individual risk taking in a time-dependent manner and leads to anti-social risk. Eur J Neurosci.

[16] Steinbeis N, Engert V, Linz R, Singer T (2015). The effects of stress and affiliation on social decision-making: investigating the tend-and-befriend pattern. Psychoneuroendocrinology.

[17] Romero-Rivas C, Rodriguez-Cuadrado S (2021). The psychological impact of the COVID-19 pandemic affected decision-making processes. Span J Psychol.

[18] Gruber J, Prinstein MJ, Clark LA (2021). Mental health and clinical psychological science in the time of COVID-19: challenges, opportunities, and a call to action. Am Psychol.

[19] Dickerson SS, Kemeny ME (2004). Acute stressors and cortisol responses: a theoretical integration and synthesis of laboratory research. Psychol Bull.

[20] Luo M, Guo L, Yu M, Jiang W, Wang H (2020). The psychological and mental impact of coronavirus disease 2019 (COVID-19) on medical staff and general public – a systematic review and meta-analysis. Psychiatry Res.

[21] Ebrahimi OV, Hoffart A, Johnson SU (2021). Physical distancing and mental health during the COVID-19 pandemic: factors associated with psychological symptoms and adherence to pandemic mitigation strategies. Clin Psychol Sci.

[22] Rudenstine S, McNeal K, Schulder T (2021). Depression and anxiety during the COVID-19 pandemic in an urban, low-income public university sample. J Trauma Stress.

[23] Lee J, Solomon M, Stead T, Kwon B, Ganti L (2021). Impact of COVID-19 on the mental health of US college students. BMC Psychol.

[24] Brand M, Labudda K, Markowitsch HJ (2006). Neuropsychological correlates of decision-making in ambiguous and risky situations. Neural Netw.

[25] Pollak Y, Dayan H, Shoham R, Berger I (2020). Predictors of non‐adherence to public health instructions during the COVID ‐19 pandemic. Psychiatry Clin Neurosci.

[26] Pfattheicher S, Nockur L, Böhm R, Sassenrath C, Petersen MB (2020). The emotional path to action: empathy promotes physical distancing and wearing of face masks during the COVID-19 pandemic. Psychol Sci.

[27] Syropoulos S, Markowitz EM (2021). Prosocial responses to COVID-19: examining the role of gratitude, fairness and legacy motives. Pers Individ Dif.

[28] Carlo G, Randall BA (2002). The development of a measure of prosocial behaviors for late adolescents. J Youth Adolesc.

[29] Carlo G, Knight GP, McGinley M, Zamboanga BL, Jarvis LH (2010). The multidimensionality of prosocial behaviors and evidence of measurement equivalence in Mexican American and European American early adolescents. J Res Adolesc.

[30] Spielberger CD, Gorsuch RL, Lushene R, Vagg PR, Jacobs GA (1983). Manual for the State-Trait Anxiety Inventory.

[31] Spielberger CD (1989). State-Trait Anxiety Inventory: Bibliography.

[32] Hamilton CM, Strader LC, Pratt JG (2011). The PhenX Toolkit: get the most from your measures. Am J Epidemiol.

[33] Bacon SL, Lavoie KL, Boyle J, Stojanovic J, Joyal-Desmarais K, iCARE study team (2021). International assessment of the link between COVID-19 related attitudes, concerns and behaviours in relation to public health policies: optimising policy strategies to improve health, economic and quality of life outcomes (the iCARE study). BMJ Open.

[34] Hayes AF (2022). Introduction to Mediation, Moderation, and Conditional Process Analysis Third Edition: A Regression-Based Approach.

[35] Gupta S, Watanabe S, Laurent SM (2021). Psychological predictors of vaccination intentions among U.S. undergraduates and online panel workers during the 2020 COVID-19 pandemic. PLoS One.

[36] Hromatko I, Tonković M, Vranic A (2021). Trust in science, perceived vulnerability to disease, and adherence to pharmacological and non-pharmacological COVID-19 recommendations. Front Psychol.

